# Strongyloides stercoralis Hyperinfection Syndrome: A Neglected Cause of Abdominal Pain

**DOI:** 10.7759/cureus.10671

**Published:** 2020-09-26

**Authors:** Paramarajan Piranavan, Pooja Kalantri, Deepali Pandey, Hariharan Sivakumar Bharadwaj, Ashish Verma

**Affiliations:** 1 Internal Medicine/Rheumatology, State University of New York Upstate Medical University, Syracuse, USA; 2 Internal Medicine, Saint Vincent Hospital, Worcester, USA; 3 Internal Medicine/Haematology and Oncology, University of Kentucky, Lexington, USA; 4 Pathology, Baystate Medical Center, Springfield, USA; 5 Nephrology, Saint Vincent Hospital, Worcester, USA

**Keywords:** strongyloides hyperinfection syndrome, rare cause of acute abdominal pain, travel, international and travel medicine

## Abstract

*Strongyloides stercoralis *infection is usually acquired from tropics or subtropics, often causes asymptomatic chronic infection, but in immunosuppressed, it can lead to hyperinfection syndrome. We report a case of chronic abdominal pain resulting from *Strongyloides *infection in a 55-year-old male with a history of partial small bowel resection for small intestinal lymphoma and a recent diagnosis of chronic kidney disease with proteinuria on steroid therapy. He presented with chronic abdominal pain, nausea, loss of appetite, and weight loss. Initial laboratory workup and imaging including retroperitoneal ultrasound and CT of the abdomen/pelvis were within normal limits, and he was discharged on acid suppression therapy. He was readmitted with worsening symptoms and underwent esophagogastroduodenoscopy (EGD) and duodenal biopsy, which revealed *Strongyloides *infection. We later discovered a travel history to Cambodia. His symptoms resolved with ivermectin therapy. This case highlights the importance of travel history, which can prevent unnecessary investigations and delay in the diagnosis.

## Introduction

*Strongyloides stercoralis* is a soil-transmitted threadworm with an estimate of 30-100 million people infected worldwide [1]. Symptoms can range from chronic clinically asymptomatic to acute symptomatic and are associated with the path of larval migration to the small intestine.A study done by Meysam et al. found that 15.7% of patients were asymptomatic and 84.3% of patients were symptomatic in chronic infection [2]. During acute infection, almost all patients are symptomatic*. *Acute symptoms are local irritation due to skin penetration by the larva, dry cough due to tracheal irritation caused by larval migration through the lungs, and gastrointestinal (GI) symptoms like abdominal pain, anorexia, diarrhea, and constipation when the larvae finally infect the small intestine. Chronic symptoms can range from clinically asymptomatic to recurrent urticaria, larval currens, rashes, pruritus ani, recurrent asthma, and chronic GI symptoms like intermittent vomiting, borborygmus, constipation, and diarrhea [3]. Unusually arthritis, nephrotic syndrome, duodenal obstruction, and chronic malabsorption have been reported [4-7]. An alteration in immune status can cause accelerated autoinfection by larvae (hyperinfection syndrome) and lead to the development or exacerbation of GI or pulmonary symptoms. The history of exposure to the parasite may be remote and increased awareness among physicians will help to explore the travel history in appropriate clinical circumstances. We hereby present a case of *Strongyloides *infection manifesting as new-onset abdominal pain and constitutional symptoms in the setting of steroid use and a remote history of partial small bowel resection due to small intestinal lymphoma.

## Case presentation

A 55-year-old Asian American male with a past medical history significant for small intestinal lymphoma status post small bowel resection and chemotherapy (currently in remission), and chronic kidney disease (CKD) stage 4, was admitted with progressively worsening epigastric abdominal pain, nausea, vomiting, poor appetite, and significant weight loss over a few weeks prior to admission. He was in his usual state of health six months ago when he noticed intermittent dry cough and fever. He was treated a few times for upper respiratory tract infection as an outpatient with antihistamines and oral antibiotics with subsequent improvement. He developed intermittent abdominal pain a few weeks prior to admission, which was attributed to suspected gastritis from nonsteroidal anti-inflammatory drugs (NSAIDs) usage. His abdominal pain did not improve with acid suppression therapy and cessation of NSAIDs. He also reported a 30-pound weight loss over the last three months. Review of systems was positive for malaise, lower limb edema, and intermittent loose stools and negative for melena, hematemesis, hematochezia, dyspnea, lymphadenopathy, or chest pain. 

He was diagnosed with small intestinal lymphoma in 1997 and was treated with partial small bowel resection and chemotherapy for six months and declared to be in remission by his oncologist. His other notable past medical histories included hypertension, hyperlipidemia, CKD stage 4, and gout. As a workup for his CKD and persistent proteinuria, he underwent renal biopsy and was diagnosed with advanced focal segmental glomerulosclerosis (FSGS), likely secondary, chronic active interstitial nephritis, superimposed with mild IgA nephropathy. He was initiated on high-dose prednisone therapy (1 mg/kg) with the aim of slow taper for his kidney disease as an outpatient a few months prior to the current presentation. 

His family history was negative for any renal disease, autoimmune diseases, malignant neoplasms, or young-onset coronary artery disease. He was a nonsmoker, and reported no alcohol usage or any other illicit drug usage. He denied any significant travel history on his initial admission.

His vitals on admission were within normal limits. Physical exam was notable for mild conjunctival pallor and mild epigastric tenderness on deep palpation without any guarding or rigidity. The abdomen was soft and bowel sounds were present in all quadrants. His rest of the physical exam was within normal limits.

His complete blood count (CBC) showed mild normocytic anemia and leukocytosis with neutrophil predominance upon admission. However, a few months prior to admission, he had persistent mild eosinophilia, which peaked up to 12% of total leukocytes with a normal total white count. However, after commencing steroids for renal disease, his eosinophilia had resolved and now developed leukocytosis with neutrophil predominance. His basic metabolic panel was consistent with mild acute kidney injury in the background of CKD and moderate hypotonic hyponatremia. His liver function tests, cardiac enzymes, and thyroid functions were within normal limits. His blood and urine cultures were negative. Iron studies were suggestive of anemia of inflammation. He had marginally elevated lipase (Tables 1, 2). His stool for cryptococcus antigen and giardiasis were negative. HIV fourth-generation testing was negative. His stool was not tested for amoeba and *Strongyloides *as the travel history could not be obtained.

**Table 1:** Laboratory results: hematology, chemistry (serum and urine) results CBC, complete blood count; BMP, basic metabolic panel; MCV, mean corpuscular volume; MCHC, mean corpuscular hemoglobin concentration; BUN, blood urea nitrogen; eGFR, estimated glomerular filtration rate

Lab	Admission		Discharge	Normal range
CBC				
WBC	11.5		5.4	3.9-11 x 1000/µL
Hemoglobin	11.7		10.6	12.5-17 g/dL
Hematocrit	34.7		31.2	36%-50%
MCV	89		90	80-100 fL
MCHC	34		34	31-36 g/dL
Platelet count	231		215	150-450 x 1000/µL
BMP				
Na		126	126	134-144 mEq/L
K		4.9	5	3.6-5.6 mEq/L
Cl		91	94	96-109 mEq/L
Bicarb		25	21	20-32 mEq/L
BUN		65	39	5-26 mg/dL
Creatinine		3.4	2.57	0.5-1.5 mg/dL
eGFR		19.2	26.9	>59 mL/min
Calcium		8.3	8.2	8.3-10 mg/dL
Blood sugar		170	145	65-99 mg/dL
Others				
Serum osmolality		287	NA	275-295 mOsm/kg
Urine osmolality		341	NA	300-900 mOsm/kg
Urine sodium		48	NA	Not defined (mEq/L)
Lipase		238	NA	0-59 U/L

**Table 2:** Laboratory results: urine analysis

Variables	Value
Urine color	Yellow
Urine appearance	Clear
Urine pH	5.5
Urine specific gravity	1.012
Urine protein	100 mg/dL (high)
Urine ketone	Negative
Urine blood	Trace
Urine nitrite	Negative
Urine bilirubin	Negative
Urine urobilinogen	0.2 (normal)
Urine leukocyte esterase	Negative
Urine glucose	Negative

Imaging

His CT abdominal and pelvis showed mild fecal loading and right renal cyst, and was negative for any GI, hepatobiliary, and pancreatic pathology that could explain the patients’ symptoms. His chest x-ray was within normal limits without any obvious infiltrates or nodules. Retroperitoneal ultrasound revealed normal renal parenchyma without any evidence of hydronephrosis.

Differential diagnosis

The differentials initially were broad and included the following.

1. Chronic GI infections or systemic infections with GI manifestations like giardiasis, cryptosporidiosis, amoeba infection, intestinal tuberculosis, and HIV, very rarely small intestinal bacterial overgrowth and Whipple's disease

2. Chronic GI conditions like gastritis, reflux disease, and irritable bowel syndrome

3. Chronic inflammatory/autoimmune diseases like celiac disease, inflammatory bowel disease

4. Neoplasms like GI gastric carcinoma, intestinal lymphoma, pancreatic or hepatobiliary neoplasms

His significant weight loss and chronic abdominal symptoms increased the suspicion for GI neoplasms rather than chronic gastritis. Celiac disease was less likely with negative tissue transglutaminase IgA antibodies. CT abdomen and pelvis did not reveal any GI neoplasms; however, it could not have been ruled out without endoscopic evaluation.

Treatment

He received intravenous fluids and his symptoms partially improved with the resolution of hypovolemia and hyponatremia. He was evaluated by the gastroenterology team with a plan for outpatient gastroenterology evaluation of his poor appetite and weight loss with EGD and colonoscopy, especially given his history of intestinal lymphoma.

Outcome and follow-up

**Figure 1:**
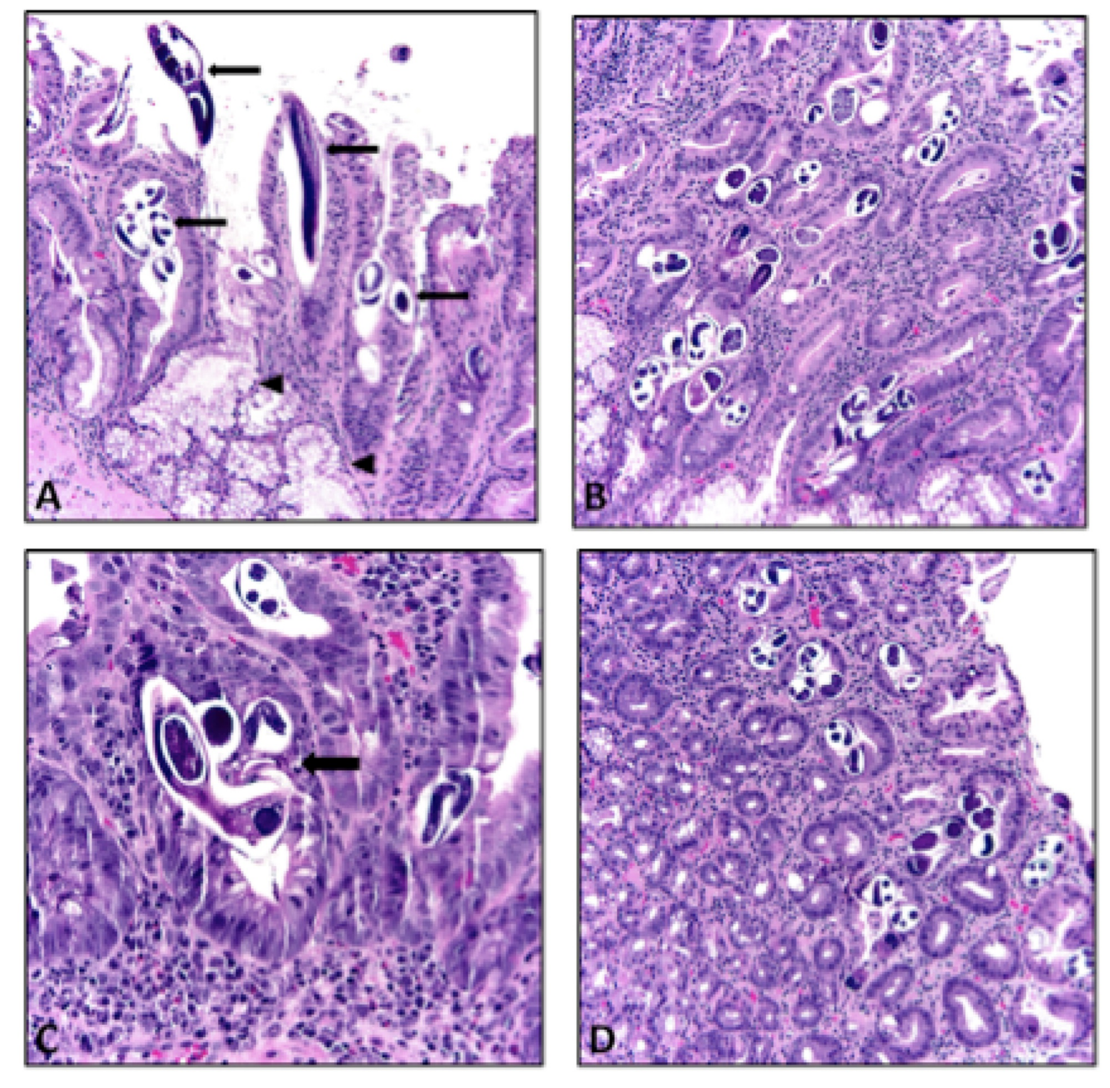
Histology of biopsy (A) Histologic section illustrating *Strongyloides *infection (arrow) of the duodenum. Brunner’s glands (arrowhead) are spared (H&E, x20). (B) This section illustrates the high parasite load noted in the duodenal mucosa (H&E, x20). (C) Histologic section shows background mucosa with dense mixed inflammatory cell infiltration, including lymphocytes, plasma cells, and prominent eosinophils. The lining epithelium shows reactive epithelial changes with regenerative crypts containing *Strongyloides *parasite (arrow) (H&E, x40). (D) Histologic section showing gastric mucosa also being involved by the parasite (H&E, x20). The background mucosa contains mixed inflammatory cell infiltration similar to that seen in the duodenum.

However, he was readmitted again before his initial outpatient follow-up, with melena and worsening abdominal symptoms. He underwent EGD that revealed a medium-sized hiatus hernia with normal-appearing mucosa, diffuse area of severe erosive gastritis, duodenitis noted in the bulb, and second and third portion of the duodenum. He underwent multiple biopsies from all the above areas. Although there was diffuse ulceration, erythema, and friable mucosa noted in the stomach and duodenum, there was not any evidence of active bleeding. Histopathology revealed numerous *Strongyloides *organisms in gastric fundic body mucosa, heavy parasite load in the duodenal mucosa, and evidence of chronic active gastritis and duodenitis with ulceration (Figure 1). Upon further questioning, the patient revealed his travel history to Cambodia to visit his family two years ago; however, on return he did not have any symptoms.

Infectious disease was consulted. Further workup including stool studies showed infestation by rhabditiform larvae of *Strongyloides stercoralis *and positive *Strongyloides *serum IgG antibodies. His steroids were discontinued. He was started on oral ivermectin therapy and continued on acid suppression therapy with proton pump inhibitors. His repeat stool studies remained negative. His previous renal biopsy was reevaluated and did not have any evidence of *Strongyloides *infection. 

## Discussion

*Strongyloides stercoralis* can undergo a free-living cycle of development as well as a parasitic cycle. It is distinguished among helminths by its unique capability to replicate in humans. Humans usually acquire *Strongyloides stercoralis* transcutaneously when filariform larvae in fecal-contaminated soil penetrate the skin or mucous membranes. The mechanism of migration to the small intestine is not clearly defined but believed to be through the pulmonary route. Organisms enter the bloodstream and are carried to the lungs, ascending the tracheobronchial tree, and then swallowed to enter the GI tract. Only adult female worms are seen in humans and subsequent reproduction occurs asexually through parthenogenesis [8]. They lay eggs that hatch into rhabditiform larvae, which are either shed in the stool or they mold into infective larvae and penetrate the intestinal wall or perianal skin and cause autoinfection that can allow them to persist in hosts for decades [8,9]. If rhabditiform larvae are shed in the stool, they under warm moist conditions found in tropical and subtropical endemic areas can either mold into infective filariform larvae or develop through succeeding rhabditiform stages into free-living adults. Sexual reproduction occurs exclusively in the free-living stage.

In the immunocompetent host, it is felt that the population of adult worms in the intestine is regulated by cellular immune effector mechanisms and intrinsic parasite biology. With an alteration in the host immune system, even one adult female can multiply rapidly by parthenogenesis, leading to accelerated autoinfection, resulting in hyperinfection and/or dissemination.

Hyperinfection, the syndrome of accelerated autoinfection, is usually the result of an alteration in immune status [10]. Larvae in non-disseminated hyperinfection are increased in number but confined to the organs normally involved in the pulmonary autoinfective cycle (i.e., GI tract, peritoneum, and lungs), whereas in disseminated infection larvae migrate to organs beyond the range of the pulmonary autoinfective cycle [1].

The clinical manifestations of *Strongyloides stercoralis* hyperinfection can vary widely. The constitutional symptoms include fatigue, weakness, and total body pain. GI manifestations can include abdominal pain, often described as cramps or bloating in nature, nausea, vomiting, anorexia, constipation, watery diarrhea, weight loss, and difficulty swallowing [11,12]. It can also manifest as GI bleeding and small bowel obstruction [13,14]. Peripheral edema or ascites can occur as a consequence of protein-losing enteropathy leading to acute or worsening hypoalbuminemia [15]. Cardiopulmonary manifestations include cough, wheezing, choking sensation, hoarseness, sore throat, chest pain, hemoptysis, palpitations, atrial fibrillation, dyspnea, and rarely, pneumothorax and respiratory collapse. Respiratory alkalosis is also common. Dermatologic manifestations include pruritic linear streaks of the lower trunk, thighs, and buttocks (larva currens), petechiae, and purpuric rashes of the same areas, vasculitis and/or associated gram-negative sepsis with disseminated intravascular coagulation. CNS symptoms include aseptic/gram-negative/eosinophilic meningitis and hyponatremia [3].

The diagnosis is based on stool studies (microscopy, agar plate culture, PCR where available) and serology (enzyme-linked immunosorbent assays, indirect immunofluorescence microscopy, gelatin particle agglutination, and immunoblot). Skin biopsy in case of dermatologic manifestations, respiratory specimens (sputum, bronchoalveolar lavage, pleural fluid) in case of pulmonary involvement, paracentesis in case of ascites, and lumbar puncture in case of meningitis, all demonstrating that the larvae are other diagnostic tools. If hyperinfection is suspected, blood cultures to rule out gram-negative (*Escherichia coli*, *Klebsiella pneumoniae*, *Proteus mirabilis*, *Pseudomonas*) and gram-positive bacteremia like* Enterococcus faecalis* should be taken. Polymicrobial infections can also occur. There is no role for routine endoscopy for diagnosis of strongyloidiasis; for patients with GI symptoms of uncertain etiology who undergo endoscopy, it may be possible to establish a diagnosis of strongyloidiasis through endoscopy [16].

Treatment with anthelminthic therapy (ivermectin) is warranted for symptomatic and asymptomatic individuals, regardless of immune status. The goal of treatment is a cure, in order to prevent the development of severe disease in the context of chronic autoinfection [17].

In our patient, with a history of travel to Cambodia, new-onset abdominal symptoms occurred after starting prednisone for his CKD and proteinuria. He had respiratory symptoms for a few months preceding the onset of his abdominal symptoms. This was symptomatically treated as a respiratory viral syndrome. This could be a respiratory manifestation of his *Strongyloides *infection and we cannot confirm it as no *Strongyloides *testing was done at that time because he did not reveal the travel history earlier. Worsening of abdominal symptoms in the setting of immunosuppression, heavy parasite load on duodenal biopsy, and presence of the parasite in the stool studies establish the diagnosis of hyperinfection syndrome. Reevaluation of the renal biopsy reports did not reveal evidence of infection, which could explain his worsening renal function and proteinuria. Typically, minimal change findings are reported on the renal biopsies in *Strongyloides *infection [5]. Our patient had the renal disease from various pathologies such as FSGS, IgA nephropathy, and interstitial nephritis. Had the travel history been known earlier, the diagnosis would not have been delayed. Once the diagnosis was established, he was successfully treated with the anthelminthic medication, ivermectin, resulting in the cure, as documented by improvement in symptoms and absence of the larvae on repeat stool cultures.

## Conclusions

*Strongyloides *is not a common cause of GI symptoms in this country. Hence, a thorough history taking regarding travel is important in selected cases to prevent the worsening of symptoms and disseminated complications. Once confirmed by stool studies demonstrating the larvae or by serology, treatment is quite simple, with ivermectin being the drug of choice, resulting in almost complete remission. Prevention of autoinfection and transmission of the disease in the community is also important, given the rarity of the disease in the nonimmigrant population. Early recognition, diagnosis, and treatment would also result in resource management, preventing unnecessary hospitalizations, and decreasing the economic burden on the healthcare systems and the patients.
